# Spinal Diseases: With an Improved Plan of Treatment. Including What Are Generally Called Nervous Complaints

**Published:** 1841-04-01

**Authors:** 


Spinal Diseases: with an improved Plan of Treatment. Including what are commonly called Nervous Complaints, and numerous Examples from upwards of 150 Cases. By *John Hey Robertson,* M.D. Octavo, pp. 160. Glasgow and London. 1841.

The object of this volume is confined to three classes of spinal affections. Pott's malady, disease of the spinal bones, ending in pressure on the spinal cord, with or without paralysis of the parts below. 2d. Lateral or serpentine curvature. 3d. Disordered condition of the spinal nerves. This last is the most common of all, and is often dependent on the lateral or angular curvature; but frequently idiopathic. It is unnecessary for us to take any notice of the anatomy, symptoms, or pathology of the first two classes of spinal affection. The third, or functional derangement of the spinal nerves occupies chiefly our author's attention. He dislikes the term "spinal irritation," as conveying no very definite meaning to his mind. He seems to think that, in all cases, there is some change of structure, though the eye or the scalpel be unable to detect it.

"Disordered action may be going on in some distant part, and this propagated up the course of the nerve, and being long-continued, may induce a condition of the nerve which will again produce or keep up this disordered action, no alteration all the while taking place in its physical condition. This I can conceive, but am of opinion, that it is far more likely, that long-continued derangement at the extremity of a nerve will end in producing actual alteration of structure in the nerve, although it may be to an extent that the eye, assisted by the knife, cannot, in our present state of knowledge, detect, or even comprehend.

In other cases again, where the mischief originates in the nerve or its coats, and is propagated to its extremity, it is highly probable, that it is induced by some change in the nerve itself, or its sheath, amounting to a degree of subacute inflammation. This I am also led to believe, from the facility with which the disease can be removed.

Again, in by far the great majority of those cases, the complaint is brought on by actual pressure upon a spinal nerve. Long-continued position, by keeping the column bent one way, presses unduly on the nerve, produces pain at its extremity, and by-and-by in its course. This pressure upon one part of a nerve, will act as pressure does in any other part of the body,—interrupt the natural action of the nerve, gall and irritate, until it become inflamed, remaining so until the cause be removed, and the effects produced subdued by the usual means.

When the spine has been allowed to recover its proper position, and no actual permanent curvature has taken place, the pain and distress produced by pressure on some of the nerves very frequently remains,—the mere removal of the cause has not been followed by the cessation of the effects; another analogical evidence that inflammatory action, or something approaching to it, has been induced in the nerve, or its sheath. Were there no other effect produced than mere interruption to the passage of the nervous fluid, the nerve should be restored to its original or sound condition on the removal of the pressure." 23.

The cure of lateral curvature must necessarily be slow; but the effects of pressure on the nerves, Dr. R. has always been able to relieve with certainty and celerity.

The third chapter of the work is dedicated to the symptomatology of spinal disorder, whether it consists in "disturbed action," irritation, or inflammation. The symptoms are numerous, and often of the most opposite kind. There is usually pain or numbness in some part of the chest or back, often about the sternum—*nervousness*—head-aches—sensation of weight or dragging, in the neck, back, or shoulders—a feeling of pricking or tingling in the limbs. Sometimes there is a sense of coldness—there is a sense of *sleeping* in the arms and legs—occasional coldness in the back, shoulders, neck, or head—oppressed breathing—tightness across the chest—palpitation—weakness of back—inability to walk far—or to sit upright or to either side, without pain. Pressure on the spine, with or without a hot sponge, will generally occasion some pain or tenderness. Cough, and pain in the chest, are not unusual, and sometimes raise apprehensions of phthisis.

"Partial or occasional blindness, or irritability of the eyes to light. Inability to lie on one side, or to bend to one side, without severe pain. Eructations, with irritability of the stomach, and vomiting.

Irritability of the bladder, with nephritic or gravelly pains stretching down the thighs. Constipation, and pains in the bowels. Pain in the right side, in the region of the liver, and also of the stomach.

*Those functions peculiar to females frequently absent, or very irregular,* and usually attended with severe pains in the lower part of the back. Limbs puffy, or swollen. Colour of the skin bad, generally dead white, or slightly tending to a faint yellow.

There is sometimes numbness, dulness, or paralysis of a part; sometimes the patient trips or stumbles on attempting to walk. There may be curvature, or projection of some part of the spine." 27.

The foregoing symptoms will not all appear in any case, but some of them in all cases.

CHAP. IV. is on the causes of these spinal affections. *Position* at school, or under tuition at home, is a fertile cause among the wealthier classes of society—and is a severe tax on opulence. The over-exertion of the brain, in modern education, is still more injurious than awkward or long-continued bad posture. In fact, the causes are numerous which lead to modern spinal affections, and many of them are totally beyond our cognizance. Among the middle and lower classes of society, the causes are equally numerous, though often of a diametrically opposite kind.

We must pass over several chapters of the work which, although they convey useful hints to the non-medical public, add nothing to the information of the professional reader. The tenth chapter opens the subject of treatment. The following passage contains the pith of the chapter.

" A patient may have imperfect or diseased action of the Spinal nerves, either with or without lateral or serpentine curvature of the spine.

There is very little difference in the mode of relief, in the first instance, in either case. The distressing symptoms produced in curvature by pressure upon the spinal nerves, are quite capable of being relieved, without waiting for the removal, or even alleviation of the curvature. As the bend is usually gradual, mere curvature would produce no inconvenience to the patient beyond personal deformity, were it not for the injury done to the spinal nerves.

I do not know that the means I have been in the habit of adopting with so much advantage, in the treatment of these affections, differ much from those in use by the profession, except, perhaps, in the time and *mode* of employing them.

Cupping, particularly what is called 'Dry Cupping,' from there being no knife used, nor blood drawn, leeches, blistering, acupuncture, friction,—simple and medicated,—properly directed exercises, and sometimes a bandage of the simplest construction, comprise about the entire of my treatment, employed and varied, of course, according to the circumstances of each case. I have now and then been compelled to have recourse to a small eschar, not in simple disturbed function of the spinal nerves, but where this was complicated with deep-seated disease, involving alteration in the structure of the parts. An issue in mere curvature, whether to remedy the curve, or the inconveniences produced by it, is the very height of folly; and yet I have more than once met with patients who had them. Issues cannot be of the slightest service to the curvature, and are not at all necessary for the symptoms induced by it." 69.

It is evident that, among these remedies, " dry-cupping" is the heroic one—and its heroism appears to depend chiefly on the manner, the sleight of hand, with which the operation is performed. Dr. R. seems to unite the " suaviter in modo" with the "fortiter in re," for he assures us that, while he is enabled to draw up the integuments so as almost to half-fill the cup, the accompanying sensation is absolutely pleasurable.* The preference which he gives to dry-cupping over the scarificator is founded on several considerations—the chief of which is the saving of blood, where the individual is weakly, which is too often the case.

The mode in which Dr. R. applies the cups is not materially different from the method used some 40 or 50 years ago.

" A little piece of folded absorbent paper is dipped into alcohol—brandy, or pyroxilic spirit, keeping the end by which you hold the paper dry. Apply this to the flame of a candle, and let it drop to the bottom of the glass to be used. Turpentine does very well, but has the inconvenience of producing much smoke when burned. The dry portion of the paper adheres to the wetted or damp bottom of the glass, and when the latter is inverted and applied to the skin, the flame remains at the point farthest from the skin, and causes no irritation from its presence. The cup adheres instantly, and may be removed easily by inserting the finger-nail under its edge, to permit the access of air. The process of merely rarefying the air by the torch, in the way done by professed cuppers, is excellent when blood is to be obtained, but besides, to be well done requiring a degree of manual dexterity not likely to be possessed in general by members of the profession, does not, even when done in the best manner, produce an

---

* " It is borne with the strongest expressions of pleasure."

effect powerful enough for my purpose in dry-cupping. Their way might now and then be adopted, especially if the patient were very thin, or the part one upon which a glass of the proper shape could not easily sit. The method described, though not perhaps the most elegant, is by far the most powerful way of producing sudden determination towards the surface, and alteration of the internal action of a part. I can, in almost any instance, in a favourable part of the body, fill the glass or tumbler nigh half-full of integument and muscle, and in *a few* instances, have seen the blood sweat through the pores of a healthy but fine skin. Large glasses are to be preferred to small where they can be used; besides being more valuable as remedial agents, they are much less inconvenient to the patient. On the chest, back, belly, or hip, where the cups have plenty of space, two or more should be put on at once, and of a size much larger than those in common use." 73.

We have long thought that dry-cupping, and counter-irritation generally, have been too much neglected in this country, where polypharmacy has been nurtured by the vile mode of remuneration by bill of drugs, like the attorney's "bill of costs."

Dr. R. does not use the common glasses—and his are of such a size as to hold from six to sixteen ounces of blood; but, "instead of the usual plain round mouths, similar to that of a tumbler or wine-glass, I have it cut out at the sides, so as to make the *mouth* of the glass a segment, more or less small, of a large circle, both sides being alike." Dr. R. considers this a great improvement, as it enables him to apply the glasses to various localities where the plain and circular cups could not be adapted. Instead of strong and expensive glasses, Dr. R. gets a few strong tumblers, of different sizes, which are shaped under his eye by the glass-cutter.

"When properly done, I have a high opinion of acupuncture, and shall give one or two, *out of a number* of cases in which it was successful. It seems most useful in those instances where the pain (without curvature,) *shifts from one part of the back to another*. How it acts it is difficult to say. Some suppose its action to be electric or galvanic, and this, for various reasons, is highly probable. On removing the needles, I have invariably observed that they were coloured deep blue, from the point upwards as far as they had been inserted, and even exhibited a degree of polarity." 85.

The work is illustrated by some 30 cases, of which we can only find room for one.

"Mrs. ———, aged twenty-four, married four years, and has one child; has been ill for five years, and complains of difficulty of breathing, so bad as to make her unable to speak, until she has rested, after having walked but a very short distance; severe beating of the heart; general weakness, great degree of nervousness; pain in the breast; severe leucorrhœa; digestion bad; bowels constipated; has been under treatment for a long time, and latterly at the sea-coast, by advice of her medical attendant.

On examining, find she is wearing a pair of steel corsets, of great strength and weight, which press upon and gall the parts under the left arm-pit, and about the right shoulder-blade. She has worn them for three years. I found she had lateral curvature towards the right side, between the shoulders, and reversed below in the loins. Her right shoulder is about two inches higher up than her left, and it was for this, she says, she was ordered the steel corsets, viz. 'to keep her left shoulder up;' hence they were made to fit pretty well up to her left arm-pit. On inquiring carefully into the progress of her complaint, particularly the elevation of one shoulder, and depression of the other; it was admitted, that during that time, and wearing those cumbrous contrivances that were to 'keep her left shoulder up,' she has been getting constantly worse. The curvature, so far from diminishing, or even remaining stationary, has been steadily increasing. She has been ailing all the while, and now is compelled to apply for relief from the consequences.

The 2d, 3d, 6th, and 12th dorsal vertebræ, I found very painful to pressure, over the spinous processes, but could not discover pain at the sides, viz. over transverse processes.

The dry cups were applied to the places, and small oiled blisters thereafter. Liquor Potassæ, and Ammoniated Tincture of Valerian, internally twice a day.

18th *August.*—It is just a week to-day since I last saw her, and the difficulty of breathing, increased action of heart, pain in the breast, and feeling of weakness, are all relieved; bowels still constipated; find the 6th dorsal vertebræ still slightly sore to pressure, and a feeling of pain is complained of in lower part of neck, when she leans forward to read. Applied the dry cups to the spot on neck, and to 6th dorsal vertebra. Opening medicine.

27th *August.*—Difficulty of breathing, and beating of heart, still more diminished; pain of neck gone; bowels yet disposed to constipation; advised moderate use of light dumb-bells, and medicated friction to back.

4th *Septewber.*—Has been to the country for the last eight days, but does not think herself any better in consequence; re-examined back, and found 2d, 3d, 11th, and 12th dorsal vertebræ slightly painful to pressure, over spinous processes; re-applied the larger cups repeatedly, and placed small blisters on these parts. Continue Ammoniated Tincture of Valerian.

17th *September.*—Much better, but has caught cold, and has cough and expectoration. Inhalation, and a cough mixture, quickly removed these. The Leucorrhœa was first relieved, and then altogether removed, by astringent washes, and the use of the precipitated carbonate of iron, made by double decomposition at the moment it was to be used, the materials being given to the patient in separate phials.

25th *October.*—Has no cough now; to-day walked some miles from the country to see me; felt a little fatigued merely, and says that when she first consulted me, two months ago, she could not walk any distance, however short, without the greatest distress.

Back all sound to pressure, except on 2d and 3d dorsal; used two dry cups, and a blister, to be put on at bed-time, the size of a penny-piece.

She has been so well since, as not to have required any treatment. She has left off the use of her absurd steel machine, and taken my simple light bandage in its stead." 104.

Dr. Robertson's work is essentially popular, and not, we imagine, designed at all for the profession. We have, therefore, taken as long a notice of it as could reasonably be expected, and we have no doubt that it will prove very useful in the channels through which it will chiefly circulate.

